# Secretion of IL-6 by fibroblasts exposed to Australian honeys involves lipopolysaccharide and is independent of floral source

**DOI:** 10.1038/s41598-022-21130-6

**Published:** 2022-10-05

**Authors:** Fraser D. Russell, Jeanne C. Visagie, Jamie L. Noll

**Affiliations:** 1 Centre for Bioinnovation, University of the Sunshine Coast, Maroochydore DC, QLD 4558 Australia; 2 School of Health and Behavioural Sciences, University of the Sunshine Coast, Maroochydore DC, QLD 4558 Australia

**Keywords:** Cell biology, Immunology

## Abstract

Honey stimulates cellular secretion of cytokines, which has been attributed to activation of lipopolysaccharide (LPS)-dependent and LPS-independent pathways. The objective of this study was to identify whether LPS is present in Australian honey samples at levels that can stimulate interleukin-6 (IL-6) secretion by fibroblasts and whether it can transduce cell signalling by activating toll-like receptor 4 (TLR4). IL-6 was measured in culture media of fibroblasts exposed to honey for 24 h. LPS was detected in a 0.125 mg/mL solution of grey ironbark honey (0.61 ± 0.05 ng/g honey). TLR4 signalling was observed in RAW264.7 macrophages that were exposed to honey and this was prevented by preincubating the honey with the LPS-neutralising agent, polymyxin B. Australian *Eucalyptus*, *Leptospermum* and *Cyathode* honeys stimulated IL-6 secretion in cultured human dermal fibroblasts. To examine whether the response was dependent on floral source, fibroblasts were exposed to four different samples of grey ironbark honey obtained from Queensland and New South Wales, Australia. The magnitude of the cytokine response to these honeys was highly varied. We conclude that Australian honeys contain endotoxin at levels that can stimulate IL-6 secretion by fibroblasts and that signalling in macrophages involves TLR4 activation. The IL-6 secretory response was independent of floral source.

## Introduction

In addition to their role in extracellular matrix production and scar tissue formation, fibroblasts release cytokines to provide regulatory control over immune cell function^1^. Dermal fibroblasts basally secrete tumour necrosis factor alpha (TNF-α), interferon gamma, interleukin-6 (IL-6) and IL-12, with elevated secretions in response to stimuli such as histamine and bacterial lipopolysaccharide (LPS)^2,3^. The release of cytokines by wound fibroblasts during inflammation leads to the activation of nearby immune cells^1^. IL-6 is a pleiotropic cytokine, with pro-inflammatory responses ascribed to activation of a trans-signalling pathway (binding of IL-6 to a soluble IL-6 receptor), and regenerative and anti-inflammatory responses arising from activation of a classic signalling pathway (binding of IL-6 to a membrane-bound IL-6 receptor)^4–6^.

Clinical trials using wound dressings impregnated with honey from European honeybees (*Apis mellifera*) have reported reduced healing times in patients with chronic wounds (ulcers) and burns, and wounds arising from surgery^7^. Benefits of honey are associated with anti-bacterial, anti-inflammatory, anti-fibrotic and antioxidant effects^8,9^. While studies have reported that honey either inhibits^10^, or stimulates cytokine secretion by cells^11–13^, the mechanism by which these effects occur is unclear. LPS has previously been detected in honey samples^12–15^. A secretory response stimulated by endotoxin within honey has been proposed by some^12^, but not by others^13,15^. Where there is evidence for a stimulatory effect on cytokine secretion, it isn’t known whether different honey varieties have differing efficacy for stimulating cytokine secretion and whether the secretory response is dependent on the floral source of the honey.

The aims of this study were to identify whether LPS is present in Australian honey samples and whether it can transduce cell signalling by activating toll-like receptor 4 (TLR4) and stimulate cell secretion of the pleotropic cytokine, IL-6. The study also examines whether cellular responses are associated with the floral source of the honeys. The study provides clarity on the role of endotoxin to this response, with elucidation of mechanism using RAW264.7 macrophages and human dermal fibroblasts.

## Results

### Honey activates TLR4 signalling in RAW 264.7 murine macrophages

**Figure 1:**
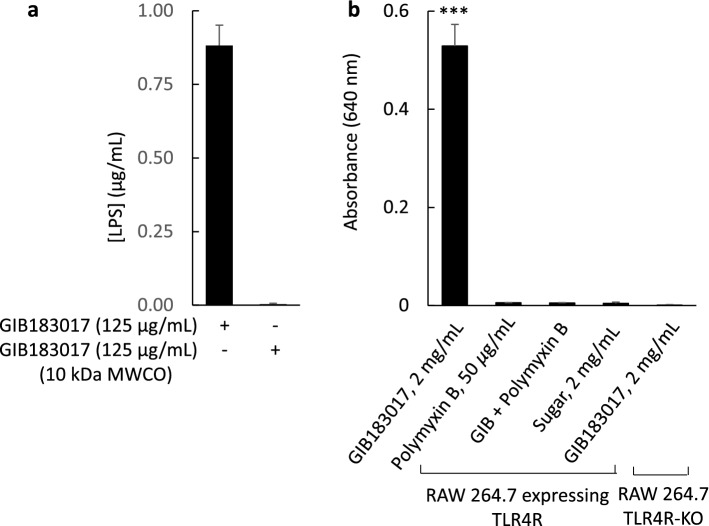
Endotoxin was detected in grey ironbark honey (183017, Table 1) using ToxinSensor™ endotoxin detection system (**a**). The endotoxin was removed by ultrafiltration using a 10 kDa molecular weight cut-off (MWCO) spin filter. The honey activated a toll-like receptor 4 (TLR4) – nuclear factor κB (NF-κB) signalling pathway in RAW 264.7 murine macrophages (**b**). Cells expressing secreted embryonic alkaline phosphatase (SEAP) formed a coloured product that was detected using colourimetry at 640 nm, after activation of TLR4. Activity was neutralised by incubation of the honey with 50 µg/mL polymyxin B for 1 h. No response to honey was detected in TLR4 knockout cells. Data are mean ± SEM; *** *p* < 0.001; (**a**) *n* = 3; (**b**) *n* = 3. Statistical analysis was performed using a one-way ANOVA.

Using an endotoxin assay, LPS was detected in a 0.125 mg/mL solution of grey ironbark honey at a concentration of 0.61 ± 0.05 ng/g honey (0.88 ng/mL, based on a density of 1.446 g/mL; *n* = 3^8^; Fig. 1a). LPS was not detected in the honey after ultra-filtration with a 10-kDa MWCO spin filter (Fig. 1a). RAW 264.7 murine macrophages were used to examine whether honey samples modulate activity via TLR4 signalling. The RAW 264.7 cells used in this assay express a knock-in reporter gene that encodes for secreted embryonic alkaline phosphatase (SEAP). When TLR4 is activated, SEAP converts a pink-coloured substrate (QUANTI-Blue™) to a blue-coloured product that is detected at 640 nm. Exposure of the macrophages to non-filtered grey ironbark honey led to coloured product formation, indicating activation of TLR4 signalling (Fig. 1b). Pre-incubation of honey with 50 µg/mL polymyxin B abolished the response. Polymyxin B alone and a sugar mixture control had no effect on TLR4 signalling. No coloured product formation was identified in cells expressing SEAP, but with knockout of the TLR4 gene, after addition of grey ironbark honey (Fig. 1b).

**Table 1:** Honey samples used in the study. NA, not applicable; QLD, Queensland; NSW, New South Wales; TAS, Tasmania.

Common name	Genus and species	Geographic region	Extraction date	Lot number
Sugar mixture	NA	NA	NA	NA
River red gum	*Eucalyptus camaldulensis*	Dalby, QLD	12/2017	181841
Grey Ironbark	*Eucalyptus paniculata*	Imbil, QLD	01/2018	183017
Grey Ironbark	*Eucalyptus paniculata*	Grafton, NSW	10/2018	190877
Grey Ironbark	*Eucalyptus paniculata*	Gympie, QLD	01/2019	192533
Grey Ironbark	*Eucalyptus paniculata*	Warwick, QLD	05/2017	174622
Messmate	*Eucalyptus obliqua*	Guyra, NSW	05/2016	176342
Yellow box	*Eucalyptus melliodora*	Emmaville, NSW	10/2015	189208
Peppermint	*Eucalyptus radiata*	Tumbarumba, NSW	02/2018	183496
Cheeseberry	*Cyathode glauca*	Triabunna, TAS	Not available	CB1
Tas. manuka	*Leptospermum scoparium*	Mawbanna, TAS	Not available	1839
NZ manuka	*Leptospermum scoparium*	New Zealand	Not available	H1905.3

### Honey and LPS stimulate secretion of IL-6 in fibroblasts

**Figure 2:**
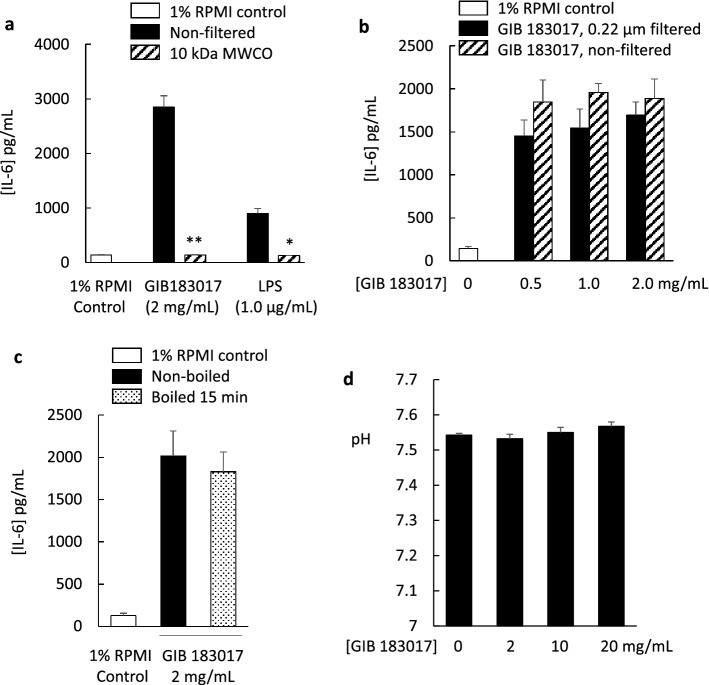
Characterisation of grey ironbark honey (183017) and lipopolysaccharide (LPS) for secretion of interleukin-6 (IL-6). Honey and LPS stimulated IL-6 secretion and this was prevented by ultrafiltration with a 10 kDa molecular weight cut-off spin filter (**a**) but not by filtration using a 0.22 µm syringe filter (**b**). The IL-6 secretory response was not affected by heat-treatment (boiled for 15 min) (**c**). pH was not appreciably affected by addition of the honey to the culture medium (**d**). Data are mean ± SEM; * *p* < 0.05, ** *p* < 0.01. (**a**) *n* = 3; (**b**) *n* = 3; (**c**) *n* = 3; (**d**) *n* = 4. Statistical analysis was performed using a Student’s *t* test (**a**–**c**) and a one-way ANOVA (**d**).

Exposure of human neonatal dermal fibroblasts to grey ironbark honey (2 mg/mL) or to 1.0 µg/mL LPS for 24 h, increased IL-6 secretion (Fig. 2a). The magnitude of the IL-6 secretory response to the honey was greater than the secretory response elicited by 1.0 µg/mL LPS. The eluants of 10 kDa MWCO spin filtration of honey and LPS samples were ineffective in eliciting an IL-6 secretory response (Fig. 2a). No significant difference in IL-6 secretion was detected between non-filtered and 0.22 µm-filtered grey ironbark honeys (Fig. 2b), or grey ironbark honey that was boiled or not boiled for 15 min (Fig. 2c). There was no appreciable change in pH following addition of the grey ironbark honey to culture medium, up to the highest concentration of 20 mg/mL (Fig. 2d).

**Figure 3:**
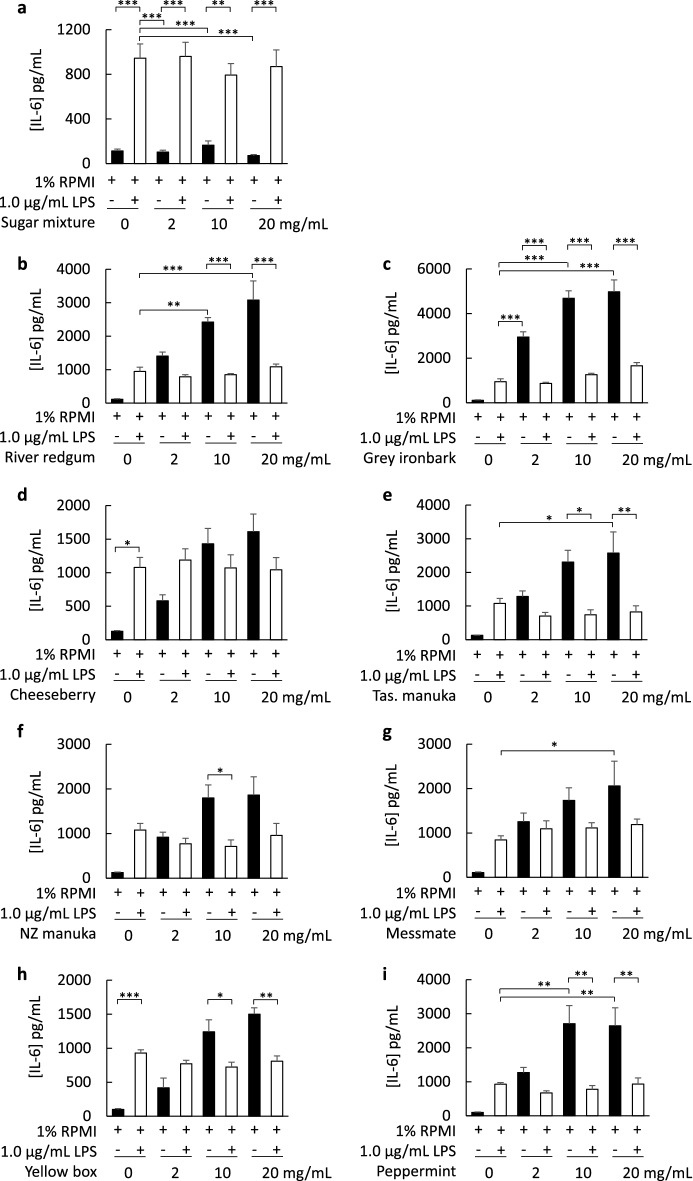
Secretion of interleukin-6 (IL-6) by human cultured neonatal fibroblasts exposed to a sugar mixture (**a**) or honey sample (**b**–**i**), in the absence or presence of 1.0 µg/mL lipopolysaccharide (LPS), for 24 h. Samples include river red gum (**b**), grey ironbark (**c**), cheeseberry (**d**), Tasmanian manuka (**e**), New Zealand manuka (**f**), messmate (**g**), yellow box (**h**) and peppermint (**i**) honeys (2–20 mg/mL). Data are mean ± SEM, *n* = 3–4; *p < 0.05; **p < 0.01; ***p < 0.001. Statistical analysis was performed using one-way ANOVA.

**Figure 4:**
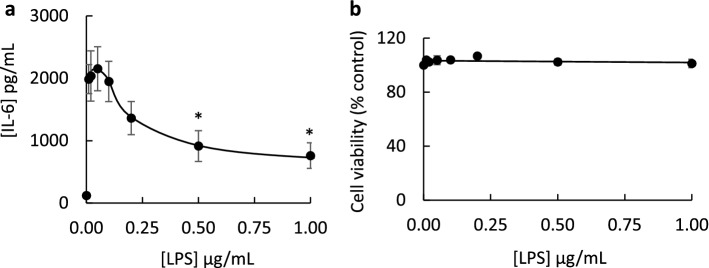
Effect of lipopolysaccharide (LPS) on IL-6 secretion by fibroblasts (**a**) and fibroblast viability (**b**). Peak IL-6 production was observed to 0.05 µg/mL LPS, with declining response at higher concentrations (**a**). Cell viability was unaffected by LPS at concentrations up to 1.0 µg/mL, determined by MTT assay (**b**). Data are mean ± SEM; * *p* < 0.05; (**a**) *n* = 3; (**b**) *n* = 4–5. Statistical analysis was performed using a Student’s *t* test.

The effect of different honey varieties (including river red gum, grey ironbark, cheeseberry, Tasmanian manuka, New Zealand manuka, messmate, yellow box and peppermint honeys) on IL-6 secretion was examined using the cultured fibroblast model. In addition to grey ironbark honey (described above), river red gum, Tasmanian manuka, messmate and peppermint honeys stimulated a greater cytokine secretory response than 1.0 µg/mL LPS (Fig. 3b,e,g,i). The capacity of most honey samples to stimulate IL-6 secretion was markedly suppressed when cells were co-incubated with honey and 1.0 µg/mL LPS (Fig. 3b,c,e,f,h,i). Cheeseberry and messmate honeys were exceptions, where the response to the honey alone was not different to honey with LPS (Fig. 3d,g). A sugar mixture control had no effect on IL-6 secretion or secretory response to LPS (Fig. 3a). To examine whether the suppressed secretory response might be attributed to the development of endotoxin tolerance over the 24 h incubation period, cells were incubated with low to high concentrations of LPS, ranging from 0.01 to 1.0 µg/mL LPS. The findings revealed a marked reduction in IL-6 secretion at the higher concentrations of LPS (0.5 µg/mL and 1.0 µg/mL) (Fig. 4a), with no reduction in cell viability (Fig. 4b). As the largest IL-6 secretion was observed in fibroblasts exposed to grey ironbark honey, this was investigated further to identify whether the response was associated with floral source.

### IL-6 secretory response to honey is independent of the floral source

**Figure 5:**
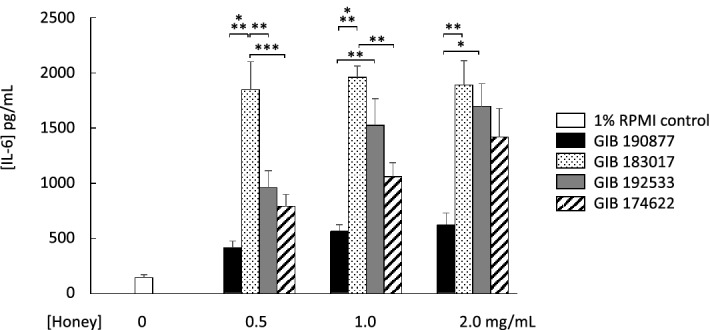
Secretory effect of four samples of grey ironbark honey, obtained from three locations in Queensland and one location in New South Wales (Table 1). The secretory response was non-uniform for this floral source of honey. Data are mean ± SEM; *p < 0.05, **p < 0.01, ***p < 0.001; *n* = 3–4. Statistical analysis was performed using a one-way ANOVA.

The effect of four samples of grey ironbark honey obtained from different regions of Queensland and New South Wales (Table 1) were compared for stimulatory effect on fibroblast IL-6 secretion. Significantly different levels of IL-6 were detected in the culture medium (range, 620 ± 107 pg/mL for 2 mg/mL grey ironbark 190877 honey to 1889 ± 222 pg/mL for 2 mg/mL grey ironbark 183017 honey; Fig. 5).

## Discussion

The aims of this study were to identify whether LPS is present in Australian honey samples and whether it can transduce cell signalling by activating TLR4 and stimulate cell secretion of the pleotropic cytokine, IL-6. The study also examines whether cellular responses are associated with the floral source of the honeys.

While the presence of LPS in honey samples has been identified previously, it is unclear as to whether the endotoxin contributes to cellular secretion of cytokines^10–14^. In the present study, we confirmed the presence of LPS in a sample of grey ironbark honey, using an endotoxin assay. As TLR4 is a sensor for LPS, we determined whether grey ironbark honey might activate TLR4 signalling. TLR4 is expressed in fibroblasts and macrophages^16^, and its activation by LPS stimulates nuclear factor-kappa B (NF-κB), which regulates inflammatory responses including induction of the gene encoding for IL-6^17^. To test this hypothesis, we used RAW 264.7 murine macrophages which express TLR4 and a knock-in reporter gene that encodes for SEAP. Grey ironbark honey stimulated SEAP activity, indicating that a constituent of the honey activates TLR4. SEAP activity was not detected in TLR4 knockout cells that were exposed to the honey. The activation of SEAP in TLR4-expressing cells was abolished when the honey was pre-incubated with polymyxin B, an antibiotic that complexes with LPS leading to its neutralisation^18^.

The molecular weight of LPS is between 10 and 20 kDa, depending on the nature of the oligosaccharide chain^19^. Using cultured fibroblasts, we showed that grey ironbark honey stimulated IL-6 secretion whereas the eluant of honey filtered through the 10 kDa MWCO filter, did not. LPS is a component of Gram-negative bacterial membranes. While filtration of the honey with a 0.22 µm syringe filter would be expected to remove bacteria, this processing did not significantly reduce honey-stimulated secretion of IL-6 by the fibroblasts, suggesting that the response is not caused by LPS that is associated with bacteria. It is possible that bacteria shed LPS and that free LPS is present in the honey. Consistent with this hypothesis, Gram-negative bacteria shed LPS^20^, and this form of the endotoxin would pass through a 0.22 µm syringe filter but be trapped by a 10 kDa MWCO spin filter.

It was of interest to note that the magnitude of the IL-6 secretory response to grey ironbark honey was greater than the peak stimulatory effect of LPS (0.05 µg/mL). We therefore don’t exclude the possibility that the grey ironbark honey contained an additional, non-LPS activator of IL-6 secretion that was also removed by ultrafiltration. Honey is composed primarily of sugars and water, and many minor constituents such as phenolic acids, flavonoids, amino acids, enzymes, minerals and vitamins that contribute to its bioactivity. Several honey constituents have been identified previously as potential stimulators of cytokine release, including LPS^12^, apisimin^21^, a 5.8 kDa molecule identified by Tonks et al.^22^ which might be apisimin^21^, type II arabinogalactan proteins^14,21^, and possibly apalbumins^22,23^, and other, as yet unidentified immunostimulatory factors^24^. The characteristics of type II arabinogalactan proteins are similar to LPS in that they are high molecular weight molecules that would be anticipated to be filtered with the 10 kDa spin filter used in this study, they are neutralised by polymyxin B and they are largely insensitive to heat treatment^14,21^. Gannabathula et al.^14^ reported a stimulatory effect of type II arabinogalactans, present in New Zealand kanuka honey, on secretion of TNF-α by phorbol-12-myristate-13-acetate-differentiated THP-1 and U937 monocytes. While we don’t exclude the possibility that non-LPS constituents of honey contribute to cytokine secretion by fibroblasts, the concentration of LPS identified in the grey ironbark honey would be sufficiently high to stimulate secretion of IL-6 by fibroblasts, as determined by our dose–response curve to LPS in these cells. The amount of LPS in the 2 mg/mL sample of honey used in the IL-6 assay is 0.014 µg/mL. Based on our dose–response curve to LPS in fibroblasts, 0.01 µg/mL LPS stimulates a near-maximal secretion of IL-6 from cultured human dermal fibroblasts (91.8% of the secretory response to 0.05 µg/mL LPS). We conclude from this that LPS is present in Australian honey and that it would stimulate IL-6 secretion by fibroblasts, at concentrations of honey used in our assay. The stimulatory factor is unlikely to be the 5.8 kDa component of manuka honey described by Tonks et al.^22^, despite it being an activator of TLR4. In that study, the stimulatory effect was heat-sensitive and resistant to neutralisation by polymyxin B whereas in this study the stimulatory effect was insensitive to heat treatment (fibroblasts) and was neutralised by polymyxin B (RAW 264.7 macrophages). It is unclear whether apalbumins can stimulate cytokine secretion^23^, or whether it associates with apisimin, which can stimulate cytokine secretion^21^.

Low pH is a known stimulator of IL-6 secretion in human esophageal epithelial (HET-1A) cells^25^. We considered the possibility that the low pH of honey might have reduced the pH of the culture medium, leading to the observed secretion of IL-6 by fibroblasts. However, we found that the buffering capacity of the culture medium was maintained as there was no appreciable change in pH following addition of the honey up to the highest concentration (20 mg/mL), thus excluding low pH as a potential confounder. Taken together, our findings indicate that the grey ironbark honey sample used in this study contains free endotoxin and that concentrations are sufficiently high to activate TLR4 signalling and stimulate IL-6 secretion.

It is not yet known whether other Australian honeys produce similar responses to grey ironbark honey and whether the effect of honey on cytokine secretion is dependent on the floral source of the honey. We therefore examined the effect of several varieties of Australian honey and a manuka honey from New Zealand, for comparison. Several of the honeys, including grey ironbark, river red gum, Tasmanian manuka, messmate and peppermint honey, stimulated significantly higher levels of IL-6 secretion compared to 1.0 µg/mL of LPS. Interestingly, the stimulatory effect was suppressed when the honey was added with LPS compared to the honey alone. This suppressed response may be because long-term exposure of cells to the high concentration of LPS can lead to endotoxin tolerance. Pre-treatment and rechallenge of cells with increasing concentrations of LPS leads to reduced cytokine secretion, which is a hallmark of endotoxin tolerance^26^. To test whether endotoxin tolerance might have developed over the 24 h incubation period, cells were incubated with LPS at concentrations ranging from 0.01 to 1.0 µg/mL. The secretory response to high concentrations of LPS was less than low concentrations, consistent with the development of endotoxin tolerance. Importantly, there was no loss in cell viability at any of the concentrations of LPS used, up to 1.0 µg/mL, thus excluding cell death as a contributing factor to the declining secretory response. Pre-treatment of RAW 264.7 macrophages with Manuka honey led to suppressed capacity of LPS to activate NF-κB, inducible nitric oxide synthase (iNOS), TNF-α and IL-1β^10^. A suppressed IL-6 secretory response was also reported in a study that incubated RAW264.7 macrophages with a combination of LPS and Thyme honey^15^. In that study, it was suggested that honey and LPS were “antagonists” of each other, and that the absence of an additive immunomodulatory response was evidence that the effect of honey was independent of its endotoxin content. However, if LPS induces endotoxin tolerance, as our findings suggest, an additive effect of honey and LPS would not be predicted. Instead, we would expect a secretory response that is no greater than to LPS alone, as observed in Fig. 3.

To examine the dependence of honey on floral source, we compared the magnitude of the IL-6 secretory response to four geographically distinct samples of grey ironbark honey. That markedly different levels of IL-6 were detected in the culture medium of cells exposed to these four samples suggests that the stimulatory effect of honey on fibroblast IL-6 secretion is independent of the floral source of the honey. Although not examined in this study, the nectar and storage compartment within the bee are potential sources of LPS. During foraging, honeybees draw nectar from flowers into their crop (foregut). The content of the foraging worker crop may be transferred to the crop of in-hive worker bees by trophallaxis. Bacterial communities are associated with floral nectar^27^, and the crop of honeybee workers, which is dominated primarily by *Lactobacillus kunkeei* (Gram positive bacteria) and Alpha 2.2 (Acetobacteraceae) (includes Gram negative bacteria)^28^. The bacterial load is not uniform amongst worker bees, with greater abundance within the crop of workers outside the hive compared to the in-hive workers^28^.

LPS-producing bacteria are a normal feature of the human gastrointestinal microbiome. Fortunately, the gastrointestinal tract provides an excellent protective barrier against gut bacteria and their associated LPS. Ingestion of honey containing LPS is therefore not hazardous and will not stimulate an immune response in humans^29^. However, the implications of applying honey to open wounds warrants further consideration. It is possible that the application of raw honey to open wounds could stimulate an immune response. Honey used in wound-healing dressings is gamma irradiated to kill potentially harmful bacteria and sterilization is achieved with a 25 kGy dose^30^. However, the efficacy of gamma irradiation to reduce LPS is uncertain^31^, with some studies reporting incomplete destruction of the endotoxin^32^. A dose of 50 kGy gamma irradiation reduced relative fluorescence signal (a marker of LPS damage) to 33% of non-irradiated LPS^32^. Consistent with the incomplete destruction of LPS, gamma irradiation doesn’t neutralise the effect of honey on cytokine secretion. Shin et al.^33^ reported a dose-dependent secretion of TNF-α and IL-10 by cultured peripheral blood mononuclear cells that were exposed to honey samples that were sterilized with 25 kGy gamma irradiation. Interestingly, LPS-induced secretion of interferon gamma, but not TNF-α, was suppressed by pre-treatment of cells with manuka and kanuka honeys. In our study, stimulated secretion of IL-6 by all honeys tested, except Cheeseberry honey, was suppressed in fibroblasts when the honey was combined with LPS.

Honey has well-described wound-healing properties that are associated with anti-bacterial effects (peroxide and non-peroxide activities; osmotic effect; low pH) and antioxidant activities. In the present study, we report the ability of LPS within honey samples to stimulate secretion of IL-6 from fibroblasts. While it is tempting to speculate that this response would lead to inflammation, IL-6 is a pleiotropic cytokine that can stimulate inflammatory responses via a trans-signalling pathway or regenerative and anti-inflammatory responses via a classic signalling pathway^4–6^. A protective effect of IL-6 in vivo was identified in a mouse model that either expresses the IL-6 gene (IL-6^+/+^ mice) or has a deleted IL-6 gene (IL-6^−/−^)^34^. In that study, the mortality rate was higher in IL-6^−/−^ mice compared to IL-6^+/+^ mice following intraperitoneal injection with 20 µg LPS per gram body weight. This finding suggests a protective effect of IL-6 in systemic inflammatory responses induced by LPS. Modelling studies predict that the classic signalling pathway is favoured over the trans-signalling pathway under conditions in which the ratio of signal transducing subunit glycoprotein 130 (gp130) to IL-6 receptor expression, is low^5^.

Identification of the floral source of honeys used in this study relied on beekeeper knowledge and is a potential limitation. Although pollen DNA metabarcoding is used to identify the primary floral source of honey, limited taxonomic resolution for Myrtaceae has been reported with this method^35^. Indeed, only two *Eucalyptus* honeys were resolved to species level using the pollen metabarcoding approach^35^, neither of which were honey varieties used in the present study.

In conclusion, the findings of this study provide evidence that Australian honey samples contain LPS and that this endotoxin can activate TLR4 signalling in cultured RAW264.7 macrophages and stimulate IL-6 secretion in human dermal fibroblasts. While a variable secretory response was identified across several honey varieties, this variability was also observed for the same honey variety (grey ironbark honey), suggesting that the response is independent of floral source. There is growing interest in the medicinal benefits of honey, with a wide range of honey-impregnated dressings on the market for facilitating wound healing. The findings of this study have implications for exposure of open wounds to raw, unprocessed honeys. Further studies comparing the wound healing potential of Australian honeys, with and without LPS, is warranted.

## Materials and methods

### Honey sample collection and processing

Honey samples were collected from Australia (Tasmania, New South Wales and Queensland) and New Zealand by beekeepers (Table 1), who also identified the floral source of the honey. A sugar control comprised a mixture of 40.2% d-fructose, 33.0% d-glucose, 7.5% d-maltose, 1.3% d-sucrose and 18% milliQ water. Honeys and the sugar control were mixed using glass rods prior to use.

Raw, unprocessed honey (0.5 g/mL) was prepared in Roswell Park Memorial Institute containing 1% heat-inactivated, 0.22 µm filtered fetal calf serum (1% RPMI-1640) medium, using 10 mL volumetric flasks. Some samples were filtered using 0.22 µm Millex®-GP syringe-driven polyethersulfone filter units (Merck Millipore Ltd., Tullagreen, County Cork, Ireland); subjected to ultrafiltration using 10 kDa molecular weight cut-off (MWCO) filters (Vivaspin® 500; Sartorius Stedim Biotech, Goettingen, Germany, Cat# VS0101; 15,000 × g at 21 °C for 5 min); boiled for 15 min; or pre-incubated with 50 µg/mL polymyxin B (Sigma-Aldrich, Castle Hill, NSW, Australia) for 1 h at 37 °C, prior to addition to the cells. pH of grey ironbark honey (183017; 5 g/10 mL) was measured using a CyberScan pH meter, Model 510 (Eutech Instruments, Singapore).

### Cell culture

NHDF-Neo human neonatal dermal fibroblasts (Lonza, Brooklyn, Victoria, Australia; Cat# CC-2509) were seeded at a density of 2.5 × 10^5^ cells per well in 24 well plates (Costar, Cat#3506). Cells were grown in RPMI-1640 media containing penicillin (100 units/mL), streptomycin (100 µg/mL), Normocin™ (100 µg/mL) and 10% heat-inactivated, 0.22 µm filtered fetal calf serum (10% RPMI-1640) for 48 h at 37 °C in a 5% CO_2_ humidified incubator. Medium was replaced with 1% RPMI-1640 medium and cells were incubated for a further 24 h. Cells were treated with LPS (0.01–1.0 µg/mL), honey (0.1–20 mg/mL) or a combination of LPS and honey, or sugar mixture (0.1–20 mg/mL), for 24 h. Culture medium was spun (10,000 × g, 5 min, 4 °C) and supernatants were collected and stored at – 80 °C until use.

RAW-Dual™ cells (InvivoGen; Cat# rawd-ismip) and RAW-Dual KO-TLR4™ cells (InvivoGen, San Diego, California, USA; Cat# rawd-kotlr4) were seeded at a density of 1.5 × 10^5^ cells per well in 24 well plates (Costar, Cat#3506). Cells were grown in Dulbecco’s Modified Eagle Medium containing penicillin (100 units/mL), streptomycin (100 µg/mL), Normocin™ (100 µg/mL) and 10% heat-inactivated, 0.22 µm filtered fetal calf serum (10% DMEM) for 48 h at 37 °C in a 5% CO_2_ humidified incubator. Medium was replaced with 1% DMEM and cells were incubated for a further 24 h. Culture media was collected, spun (10,000 × g, 5 min, 4 °C) and supernatants were used immediately, or stored at – 80 °C until use.

### Endotoxin detection

Grey ironbark honey (GIB 183017; 0.5 g/mL) was prepared in pyrogen-free water and adjusted to pH 7.20 using 5 M NaOH. The sample was diluted to 125 µg/mL using pyrogen-free water. Grey ironbark honey (125 µg/mL), with or without 10 kDa MWCO ultrafiltration, was assessed for the presence of endotoxin using a ToxinSensor™ endotoxin assay kit (GenScript USA Inc., Piscataway, NJ, USA, Cat# L00350), as per manufacturer’s instructions for chromogenic analysis. Honey endotoxin levels were compared to an LPS standard (0.25–2.0 µg/mL). Samples were aliquoted into a 96 well plate and absorbance was read at 545 nm using the EnSpire multimodal plate reader.

### Toll-like receptor 4 (TLR4) signalling

RAW-Dual™ cells and RAW-Dual KO-TLR4™ cells (InvivoGen, San Diego, CA, USA) were incubated for 24 h with grey ironbark (183017) honey that had been pre-incubated with or without 50 µg/mL polymyxin B for 1 h. A 20 µL sample of the culture medium was incubated with 180 µL of QUANTI-Blue™ reagent for 2 h at 37 °C. Solution was aliquoted to a 96 well plate and read at 640 nm. Absorbance for controls (cells incubated with 1% DMEM) was subtracted from all other readings.

### Enzyme-linked immunosorbent assay (ELISA)

IL-6 concentration in culture supernatants was measured using human IL-6 uncoated ELISA kits (Invitrogen, Tullamarine, Victoria, Australia, Cat# 88-7066), as per manufacturer’s instructions. Honey samples were diluted 20-fold to ensure readings were taken from within the range of absorbance values formed by an IL-6 standard curve. A correction factor for dilution was included in the calculations for determination of IL-6 concentration. Absorbance was read at 450 nm and 570 nm, with the latter absorbance reading subtracted from the former.

### Cell viability assay

Following removal of the culture supernatant, cells were rinsed in phosphate buffered saline (PBS) before adding 0.5 mg/mL MTT reagent (3-(4,5-Dimethyl-2-thiazolyl)-2,5-diphenyl-2H-tetrazolium bromide), prepared in PBS. Cells were incubated for 2 h at 37 °C in a 5% CO_2_ incubator before adding solubilisation solution (10% sodium dodecyl sulfate solution prepared in milliQ water and containing 0.01 M HCl). Following an overnight incubation at 37 °C, samples were mixed by pipetting the solution up and down several times. Aliquots (200 µL) were added to high binding polystyrene 96 well plates (Corning, Cat#9018). A 1:1 (v/v) sample of MTT reagent and solubilisation solution mixture, without cells, was used as a control. Absorbance was read at a wavelength of 570 nm using an EnSpire multimodal plate reader (Perkin Elmer, Glen Waverley, Victoria, Australia). Absorbance for the control was subtracted from all other readings and data were expressed as a percentage of the 1% RPMI-1640 media control. Cells were also photographed using a Nikon Eclipse Ti phase contrast microscope with a 20 × Nikon phase contrast objective lens and Nikon DS-Qi1Mc camera attachment.

### Statistical analysis

Replicates (*n*) from each experiment were analysed using SPSS Statistics Version 28.0.1, IBM and Microsoft Excel 2016. Normality distribution and equality of variance were determined using the Shapiro–Wilk test and Levene statistic, respectively. Statistical analysis was performed using one-way analysis of variance (ANOVA) with Tukey’s multiple comparisons test and Student *t*-test. A *p*-value < 0.05 was considered statistically significant, and indicated with an asterisk (*, *p* < 0.05; **, *p* < 0.01; ***, *p* < 0.001).

## Data Availability

The data that support the findings of this study are openly available in “figshare”, https://figshare.com/articles/dataset/Honey_endotoxin/20001701.

## Abbreviations

DMEM: Dulbecco’s Modified Eagle Medium
ELISA: Enzyme-linked immunosorbent assay
IL-6: Interleukin-6
LPS: Lipopolysaccharide
MWCO: Molecular weight cut-off
MTT: (3-(4,5-Dimethyl-2-thiazolyl)-2,5-diphenyl-2H-tetrazolium bromide)
RPMI-1640: Roswell Park Memorial Institute-1640
SEAP: Secreted embryonic alkaline phosphatase
TLR4: Toll like receptor 4
